# Ovarian Function, Not Age, Predicts the Benefit from Ovarian Suppression or Ablation for Premenopausal Women with Breast Cancer

**DOI:** 10.1371/journal.pone.0148849

**Published:** 2016-02-11

**Authors:** Cong Xue, Roujun Peng, Ye Cao, Shusen Wang, Yanxia Shi, Xin An, Fei Xu, Zhongyu Yuan

**Affiliations:** 1 Department of medical oncology, Sun Yat-Sen University Cancer Center, State Key Laboratory of Oncology in South China, Collaborative innovation center for Cancer Medicine, 651 Dongfeng East Road, Guangzhou, People’s Republic of China; 2 Department of GCP, Sun Yat-Sen University Cancer Center, State Key Laboratory of Oncology in South China, Collaborative innovation center for Cancer Medicine, 651 Dongfeng East Road, Guangzhou, People’s Republic of China; University of Texas Health Science Center, UNITED STATES

## Abstract

The role of adjuvant ovarian suppression or ablation (OS/OA) in premenopausal women with hormone receptor-positive breast cancer remains controversial. The purpose of our study was to examine which patients might benefit from the addition of OS/OA to tamoxifen. We analyzed the data of 2065 premenopausal patients with hormone receptor-positive invasive ductal carcinomas who were treated at Sun Yat-Sen University Cancer Center from 2000 to 2008. The five-year disease-free survival rate (DFSR) and overall survival rate (OSR) were compared by menstrual status and treatment. Compared with patients older than forty years of age, patients younger than forty years old had significant lower DFSRs and OSRs. The addition of OS/OA to tamoxifen increased the DFSR and OSR of patients with normal menstrual cycles after chemotherapy, regardless of their age at diagnosis. Patients with normal menstrual cycles after chemotherapy are the main beneficiaries of an adjuvant OS/OA.

## Introduction

Tamoxifen (TAM) has become the gold standard in adjuvant endocrine therapy for premenopausal women with Hormone Receptor (HR)-positive breast cancer [1]. In young women, especially in those younger than thirty-five years of age, although adjuvant chemotherapy and tamoxifen decreases the risk of recurrence and death, more so than in older patients, the risk is still quite high. Additional endocrine therapies are strongly recommended for these patients, for instance ovarian suppression or ablation (OS/OA) [2, 3].

Although previous studies have produced inconsistent results for adjuvant OS/OA in early stage HR-positive breast cancer patients [4–6], there it is suggested that the addition of OS/OA to chemotherapy may be beneficial to the younger patients (less than forty years old). They are most likely to have spontaneous return of the ovarian function [7, 8]. Resumption or persistence of the ovarian function after chemotherapy, presented as normal or re-attained menstruation cycle, is a poor prognostic factor in premenopausal women with HR-positive breast cancer [9–11]. Sixty to ninety percent of women younger than forty years have recovered their menstruation cycle after chemotherapy, compared to less than forty percent of those older than forty years—most of the older women can expect permanent or prolonged menstrual dysfunction [12]. This observation suggests that OS/OA greatly impacts the ovarian function in women younger than forty years, thus improving their survival chances; while additional OS/OA does not increase the survival chances of patients older than forty years of age, whose ovarian function have been damaged by the chemotherapy. Hence, we hypothesized that, instead of the age, the resumption of the ovarian function in the younger patients increased their risk of recurrence and death. The population with a normal menstrual cycle after chemotherapy, in other words with recovered ovarian function, did benefit from OS/OA.

We performed a retrospective study to examine the relationship between OS/OA, menstruation status after chemotherapy, and the therapy outcome in premenopausal patients with operable HR-positive breast cancer. We hypothesized that, regardless of their age, it was patients with normal menstrual cycles after chemotherapy who needed OS/OA. Age-specific subgroup analysis was also performed to establish the effect of OS/OA on patients with functional ovaries after chemotherapy and tamoxifen.

## Patients and Methods

### Study population

**Fig 1:**
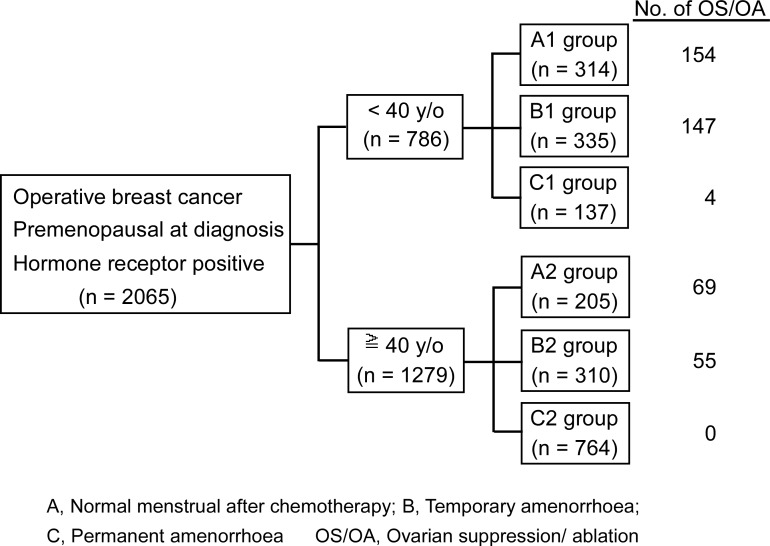
Criteria for inclusion of patients in the study.

The data of 6780 eligible patients with invasive ductal carcinoma who were treated in Sun Yat-Sen University Cancer Center between January 2000 and December 2008 were retrospectively analyzed. The exclusion criteria included: (i) metastatic disease at diagnosis (*N* = 314); (ii) postmenopausal (*N* = 2968); (iii) negative estrogen receptor (ER) and/or progesterone receptor (PR) status (*N* = 1044); (iv) no information on ER or PR status (*N* = 320); and (v) incomplete medical record (*N* = 69) (Fig 1). In total, 2065 premenopausal patients with operable HR-positive breast cancer were included in this study.

### Ethics Statement

We did not provide a specific written or verbal informed consent to the participators because it was a retrospective study. No patient identification data was collected. Moreover it was a descriptive study. We did not have any interventions afterwards. The study was reviewed and approved by the Institutional Review Board and academic committee of the Sun Yat-Sen University Cancer Center (No.B2013-028-01). Patient records/information was anonymized and de-identified prior to analysis.

### Definition of ovarian function

Patients were defined as premenopausal if their last normal menstruation happened within 6 months at diagnosis. The information about menstruation status was recorded every month during chemotherapy, radiotherapy and the treatment of tamoxifen. Amenorrhoea was defined as no menstruation for more than three consecutive months, irrespective of resumption of menstruation afterwards[13, 14]. Relatively normal menstruation was defined as menstruation lasting at least three consecutive months. OS was performed through luteinizing hormone-releasing hormone (LHRH) agonist. OA was performed through oophorectomy.

### Statistical Analysis

We divided the population into two groups; group of patients younger than forty years of age (<40 y/o) and group of patients older than forty years of age (≥40 y/o). Because most women older than forty years of age treated with chemotherapy suffered irreversible ovary damage, and there appeared to be a benefit for the use of OS/OA after chemotherapy for women younger than forty years of age [7, 15, 16]. The primary end point of this study was the five year disease-free survival rate (DFSR) between OS/OA plus TAM versus TAM in premenopausal breast cancer patients. Disease-free survival was defined as the interval from the first treatment for breast cancer to the first recurrence (locoregional relapse, distant metastasis, or contralateral breast cancer). Overall survival was calculated as the period from the date of diagnosis to the date of death from any cause or the date of the last follow-up. Clinico-pathologic parameters were assessed with the chi-square test. Cumulative survival probabilities were calculated with the Kaplan-Meier method. Survival rates were compared using the log-rank test. Multivariate analyses were performed with the Cox regression model. Some traditional prognostic factors such as age at diagnosis, tumor size, lymph node involvement, stage, ER/PR status, HER2 status, and lymphovascular invasion (LVI) were included in the multivariate analysis with enter model. Hazard ratios (HRs) were presented with their 95% confidence intervals (CIs). All statistical tests were two-tailed, and P < 0.05 was considered as statistically significant. Statistical analysis was performed with SPSS 16.0 (Chicago, IL, USA).

## Results

### Patient characteristics

Table 1 summarizes the clinico-pathologic characteristics and treatments of the 2065 patients. 786 (38.1%) of those patients were younger (<40 y/o) and 1279 (61.9%) older than forty years. The group of younger patients (<40 y/o) had tumors with higher T stage, higher lymph node positivity, higher histologic grade, and more LVI, compared with older patients (all *P*<0.05). The ER and PR expressions and the HER2 statuses are similar between two groups. The proportion of patients who underwent breast conservation surgery, adjuvant chemotherapy, and radiotherapy were higher in the younger group (<40 y/o) than in the older group (≥40 y/o) (all *P* <0.05). All patients received tamoxifen as basic endocrine therapy; the proportion of patients who received adjuvant OS/OA was greater in the younger group (<40 y/o) than in the older group (≥40 y/o) (*P* <0.001). The amenorrhoea situation was significantly different between two groups. Patients in the younger group (<40 y/o) were more likely to have normal menstruation, while patients older than forty years of age experienced much more often amenorrhea after chemotherapy (*P* <0.001). The two groups were further divided into two subgroups, according to their menstruation status after chemotherapy: group with normal menstruation (normal menses) and another group with amenorrhea (amenorrhea).

**Table 1:** Clinical characteristics and treatment of different age-specific groups.

Variable	<40 y/o	≥40 y/o	P value
**No. (%)**	786 (38.1)	1279 (61.9)	—
**Age (years)**			
**Mean ± SD**	34.5 ± 3.7	47.3 ± 10.7	—.
**Tumor size, n (%)**			
**≤2.0 cm**	217 (27.6)	410 (32.1)	0.048
**2.0–5.0 cm**	509 (64.8)	794 (62.1)	
**>5.0 cm**	60 (7.6)	75 (5.9)	
**Lymph node status, n (%)**			
**0**	350 (44.5)	684 (53.5)	0.001
**1–3**	219 (27.9)	324 (25.3)	
**4–9**	129 (16.4)	165 (12.9)	
**>10**	88 (11.2)	106 (8.3)	
**Hormonal receptor status, n (%)**			
**ER +**	642 (81.7)	1048 (81.9)	0.437
**PR +**	725 (92.2)	1171 (91.6)	
**HR+/PR+**	581 (73.9)	940 (73.5)	
**HER2 status, n (%)**			
**Positive**	128 (16.3)	200 (15.6)	0.889
**Negative**	580 (73.8)	956 (74.7)	
**Unknown**	78 (9.9)	123 (9.6)	
**Histologic grade, n (%)**			
**Ⅰ**	106 (13.5)	228 (17.8)	0.018
**Ⅱ**	301 (38.3)	494 (38.6)	
**Ⅲ**	379 (48.2)	557 (43.5)	
**LVI, n (%)**			
**Yes**	136 (17.3)	175 (13.7)	0.015
**No**	650 (82.7)	1104 (86.3)	
**Primary surgery, n (%)**			
**Mastectomy**	711 (90.5)	1190 (93.0)	0.022
**BCS**	75 (9.5)	89 (7.0)	
**Adjuvant chemotherapy, n (%)**			
**Yes**	761 (96.8)	1203 (94.1)	0.003
**No**	25 (3.2)	76 (5.9)	
**Adjuvant radiotherapy, n (%)**			
**Yes**	335 (42.6)	424 (33.2)	0.001
**No**	451 (57.4)	855 (66.8)	
**Adjuvant endocrine therapy, n (%)**			
**Tamoxifen alone**	481 (61.2)	1155 (90.3)	< 0.001
**Tamoxifen+OS/OA**	305 (38.8)	124 (9.7)	
**Amenorrhoea**			
**No**	314 (39.9)	205 (16.0)	< 0.001
**Yes**	472 (60.1)	1074 (84.0)	

SD, standard deviation; ER, estrogen receptor; PR, progesterone receptor; BCS, breast-conserving surgery; LVI, lymphovascular invasion; OS/OA, ovarian suppression/ ablation

### Survival in the four subgroups

Up to the end of December 31, 2013, the median follow-up time was 83 months (5–144 months). At the last follow-up, 374 patients relapsed, and 309 patients died.

**Fig 2:**
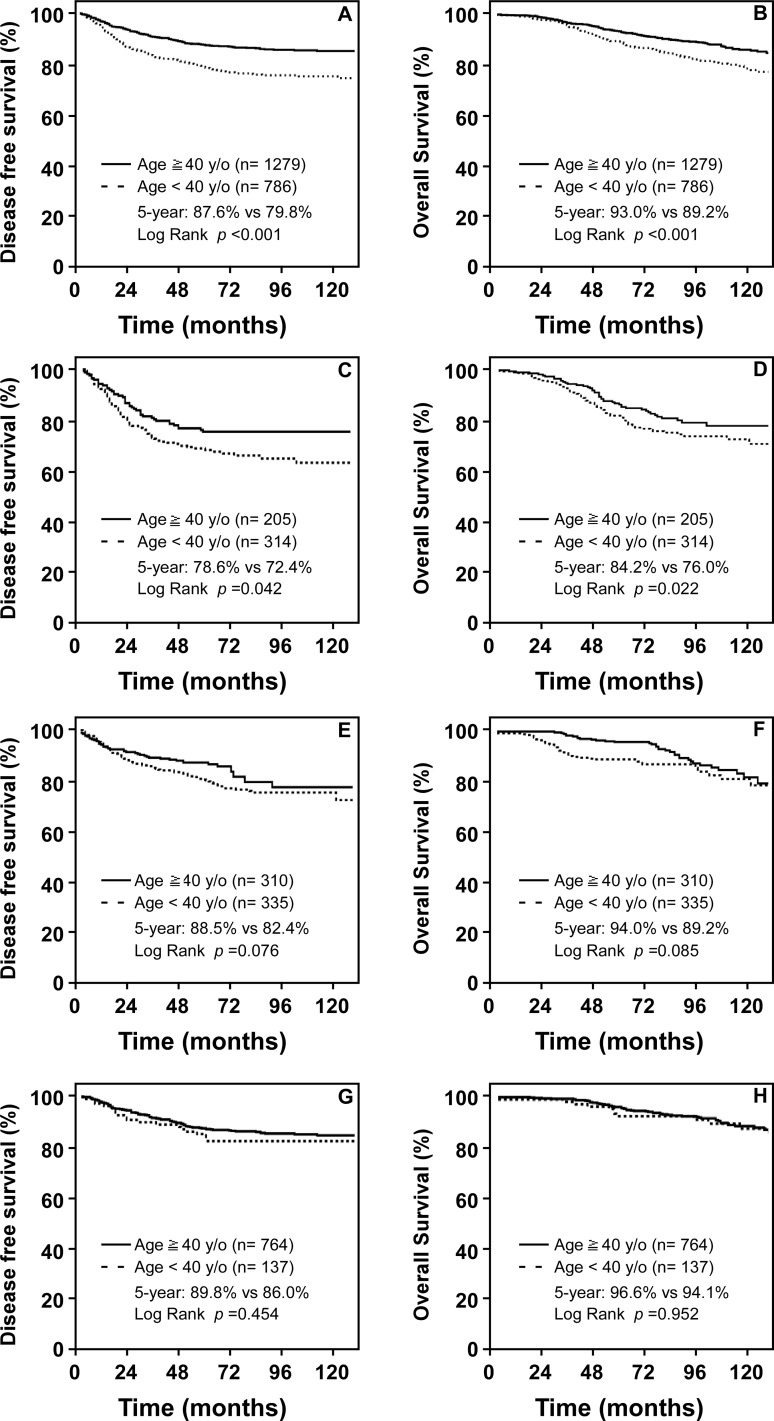
Survival curves by menstrual status after chemotherapy. Groups were divided by the age (< 40 y/o vs ≥40 y/o). (A) and (B): DFS and OS for all population; (C) and (D), DFS and OS for patients with normal menses; (E) and (F), DFS and OS for patients with amenorrhoea.

The five-year DFSR was 79.8% in the younger group (<40 y/o), it was significantly lower than in the older group (≥40 y/o; five-year DFSR = 87.6%, *P* <0.001; Fig 2A). In subset analysis, a similar difference in the five-year DFSR was observed in patients with normal menstruation (<40y/o vs ≥40 y/o: 78.6% vs 72.4%, *P* = 0.042; Fig 2C) and patients with amenorrhoea (<40y/o vs ≥40 y/o: 87.3% vs 83.4%, *P* = 0.02; Fig 2E).

The differences in overall survival rate (OSR) were observed in the total patient population, as well as in patients with normal menstruation. The five-year OSR was 93.0% in older group (≥40 y/o) and 89.2% in younger group (<40 y/o; *P* <0.001; Fig 2B). A similar difference in the five-year OSR was observed in subgroup of patients with normal menstruation (≥40 y/o vs <40y/o: 84.2% vs 76.0%, *P* = 0.022; Fig 2D). However, the difference in five-year OSRs were not detected in the subgroup of patients with amenorrhoea (≥40 y/o vs <40y/o: 93.6% vs 90.2%, *P* = 0.273; Fig 2F).

### Effects of OS/OA on survival according to menstruation status

In order to assess the role of OS/OA in specific age or menstruation status groups, we conducted another subset analysis regarding OS/OA usage by patients with normal menstruation (≥40 y/o + normal menses vs <40 y/o + normal menses) and amenorrhoea (≥40 y/o + amenorrhoea vs <40 y/o + amenorrhoea).

**Fig 3:**
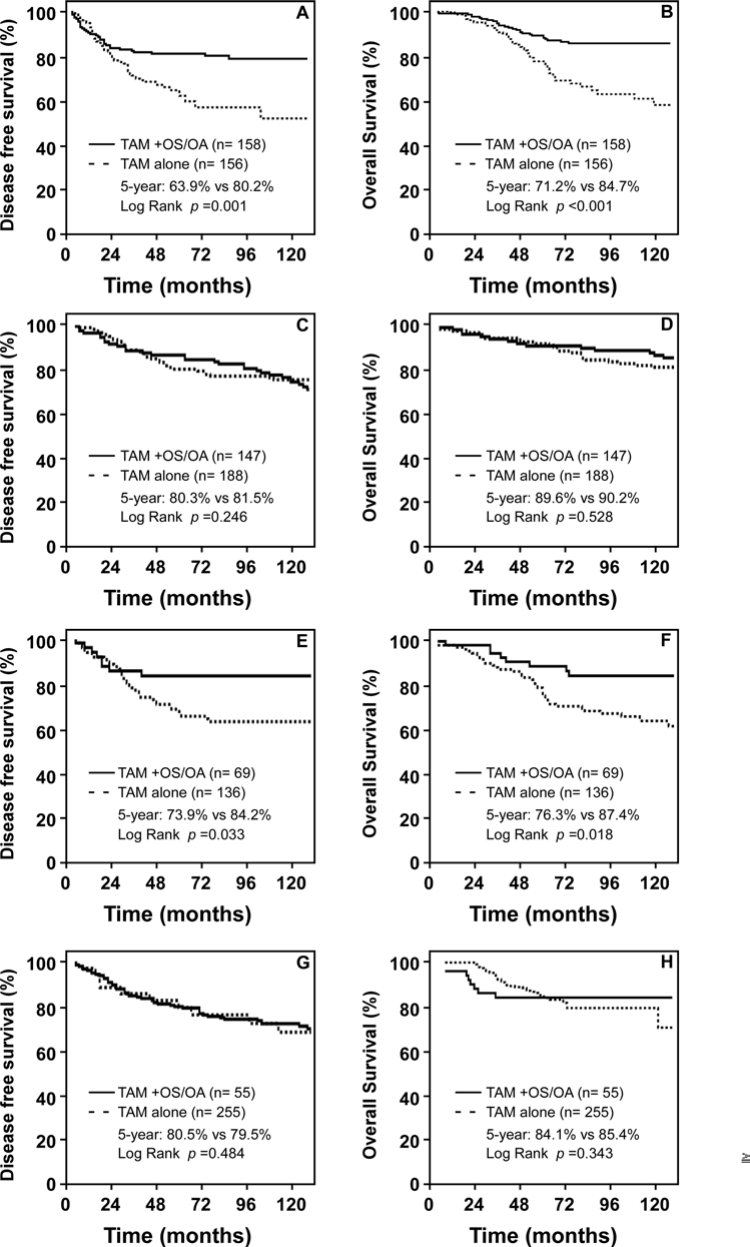
Survival curves by endocrine therapy (tamoxifen vs. tamoxifen + OS/OA). (A) and (B), DFS and OS of patients < 40 y/o + normal menses; (C) and (D), DFS and OS of patients < 40 y/o + amenorrhoea; (E) and (F), DFS and OS of patients ≥ 40 y/o + normal menses; G and H, DFS and OS of patients ≥ 40 y/o + amenorrhoea.

As shown in Fig 3A and 3E, in the population with normal menstruation, regardless of the age of the patients, the five-year DFSR was significantly better in patients who received tamoxifen and OS/OA, compared with those who had tamoxifen only. In patients <40y/o + normal menses, the five-year DFSR was 80.2% in patients who were administered tamoxifen and OS/OA, which was significantly lower in patients with tamoxifen only (five-year DFSR = 63.9%, *P* = 0.001; Fig 3A). A similar difference in the five-year DFSR was observed in patients ≥40 y/o + normal menses (TAM+OS/OA vs. TAM only: 84.2% vs. 73.9%, *P* = 0.033; Fig 3E). The difference between the effects of the two treatments (TAM+OS/OA vs. TAM only) on the values of the five-year OSRs was similar to their difference regarding the values of the five-year DSFRs (<40y/o + normal menses: TAM+OS/OA vs. TAM only: 84.7% vs. 71.2%, *P* <0.001; ≥40y/o + normal menses: TAM+OS/OA vs. TAM only: 87.4% vs. 76.3%, *P* = 0.018. Fig 3B and 3F).

In the patients with amenorrhoea, regardless of their age, there was no statistical difference in the five-year DFSRs between patients who were given tamoxifen only and those who received OS/OA in addition to tamoxifen (<40y/o + amenorrhoea: TAM+OS/OA vs. TAM only: 81.5% vs. 80.3%, *P* = 0.246; ≥40y/o + amenorrhoea: TAM+OS/OA vs. TAM only: 79.5% vs. 87.7%, *P* = 0.054. Fig 3C and 3G). Moreover, in those patients ≥40 y/o + amenorrhoea, patients with TAM+OS/OA had lower survival rates than those with TAM only. Whether the difference was statistically significant could not be established because there were very few patients older than forty years of age with amenorrhoea who received TAM+OS/OA (*N* = 55), much fewer than patients who were given TAM only (*N* = 1019). The difference between the effects of the two treatments (TAM+OS/OA vs. TAM only) on the values of the five-year OSRs was also not statistically significant. (<40 y/o + amenorrhoea: TAM+OS/OA vs. TAM only: 90.2% vs. 89.6%, *P* = 0.528; ≥40y/o + amenorrhoea: TAM+OS/OA vs. TAM only: 86.1% vs. 92.4%, *P* = 0.077. Fig 3D and 3H).

### Multivariate analysis

Multivariable analyses confirmed the descriptive results that a lower age (<40 y/o), a large tumor size(>2cm), lymph node positive status, Her2/neu positive status, histologic grade III, and lymphovascular invasion were all adverse prognostic factors for the five-year DFSR and OSR (for all factors *P* <0.05) (Table 2).

**Table 2:** Multivariate analysis of disease-free survival and overall survival in all population.

Variables	Disease-free survival			Over survival		
	HR	95% CI	P-value	HR	95% CI	P-value
**Age, years (< 40 vs ≥40)**	0.74	0.60–0.92	0.006	0.72	0.58–0.89	0.002
**Tumor size, cm (≤ 2 vs>2)**	2.43	1.64–2.30	< 0.001	2.23	1.61–3.10	< 0.001
**Node status (Neg. vs Pos.)**	1.91	1.80–3.29	< 0.001	1.49	1.14–1.95	0.003
**HER-2 status (Neg. vs Pos.)**	1.64	1.44–1.87	< 0.001	1.09	1.04–1.14	< 0.001
**Histologic grade (Ⅰ/Ⅱvs Ⅲ)**	2.18	1.51–3.02	< 0.001	1.72	1.31–2.35	0.001
**Vascular invasion (No vs Yes)**	1.83	1.37–2.43	< 0.001	2.12	1.68–2.88	< 0.001
**Menstrual status (Normal vs Amenorrhoea)**	0.68	0.54–0.85	0.001	0.41	0.32–0.52	< 0.001

Note: HER-2, human epidermal growth factor receptor 2; Neg, negative; Pos, positive; HR, Hazard ratio; HR and 95% CIs were calculated using COX regression analysis.

The results of multivariate analysis show that amenorrhoea after chemotherapy was an independent and good predictor for the five-year DFSR and OSR. Women who had experienced amenorrhoea had better DFSR (HR = 0.68, 95% CI 0.54–0.85, *P* = 0.001) and OSR (HR = 0.41, 95% CI 0.32–0.52, *P* <0.001) compared with those with normal menstruation.

After adjusting the traditional prognostic factors (age, tumor size, lymph node involvement, stage, ER/PR status, HER2 status, and LVI), OS/OA combined with tamoxifen reduces significantly the risk of recurrence (HR = 0.41, 95% CI 0.28–0.61, *P* <0.001) and death (HR = 0.33, 95% CI 0.22–0.50, *P* <0.001), for patients with normal menses after chemotherapy. However, OS/OA combined with tamoxifen does not reduce the risk of recurrence (HR = 1.16, 95% CI 0.80–1.69, *P* = 0.445), and death (HR = 0.95, 95% CI 0.61–1.48, *P* = 0.819), for patients with amenorrhoea after chemotherapy (Table 3).

**Table 3:** Multivariate analysis of disease-free survival and overall survival in patients with OS/OA or not.

Variables	Disease-free survival			Over survival		
	HR	95% CI	P-value	HR	95% CI	P-value
**Normal Menses (OS/OA+TAM vs TAM)**	0.41	0.28–0.61	<0.001	0.33	0.22–0.50	< 0.001
**Amenorrhoea (OS/OA+TAM vs TAM)**	1.16	0.80–1.69	0.445	0.95	0.61–1.48	0.819

Note: OS, ovarian suppression; OA, ovarian ablation; TAM, tamoxifen.

## Discussion

Our study showed, in line with former studies, that patients older than forty years of age had better survival chances than patients younger than forty years of age, regardless of their menstrual statuses. This meant that women younger than forty years of age, even with amenorrhoea, still had a greater risk of recurrence as they had more often larger tumors, more LVI, and other poor prognostic factors. Therefore, young patients are recommended to receive OS/OA in addition to tamoxifen [7, 8]. However, our study revealed that, rather than age, the resumption of the ovarian function after chemotherapy was the indication for OS/OA. Patients younger than forty years of age did not benefit from OS/OA in case of amenorrhoea, while women older than forty years of age who resumed their menstruations after adjuvant chemotherapy would have better survival chances if they received additional OS/OA. The purpose of OS/OA is to terminate the ovary function. The improved survivals of patients with normal menses after chemotherapy are due to the disturbance of ovary function by OS/OA. However for the patients with amenorrhea, the ovary function of patients had been damaged by chemotherapy already. Therefore, adding additional OS/OA to breast cancer patients with amenorrhea will not improve survivals.

As far as previous studies on adjuvant OS/OA are concerned, we think that the inconsistent definition of “premenopausal” led to the heterogeneous populations of these studies [4–6, 17]. Most of the prior studies used age as enrolling criterion instead of menstruation status, and the ages ranged from thirty-five to fifty years. However, there is a very large variation in ovarian function between women of the same age. A previous study showed that the incidence of women younger than forty years of age experiencing resumption of their menstruation after amenorrhoea is 27% [18]. On the other hand, the dynamic detection of sex hormone markers such as estradiol (E2) and follicle stimulating hormone (FSH) is unable to predict amenorrhoea and ovarian function recovery either [19–21]. In addition to the diverse laboratory standards and cut-offs, the employment of different markers and definitions of “clinical menses status” always led to confusing results. We believe that normal ovarian function after chemotherapy is an indication for OS/OA in addition to tamoxifen in the treatment of premenopausal women with HR positive breast cancer.

This study had several limitations. Firstly, it was a retrospective analysis, and bias might exist in reviewing data. Secondly, chemotherapy regimens were not collected in our study, which might partly affect the incidence of amenorrhoea. Thirdly, the definition of “amenorrhoea” was not uniform in the literature [10, 11]. Some revealed that amenorrhoea lasting six month was not associated with the prognosis, but this time period was not analyzed in our study. There is no consensus regarding the clinical significance of the duration of amenorrhoea. Further analysis is needed.

## Conclusion

In conclusion, the recovery of the ovarian function after chemotherapy is the only indication for an adjuvant OS/OA in premenopausal patients with HR positive breast cancer. Further study is needed to confirm the indication of OS/OA (in addition to tamoxifen) in patients with retained menstruation after chemotherapy.
